# Lipid droplet evolution gives insight into polyaneuploid cancer cell lipid droplet functions

**DOI:** 10.1007/s12032-021-01584-w

**Published:** 2021-09-28

**Authors:** Laurie G. Kostecka, Kenneth J. Pienta, Sarah R. Amend

**Affiliations:** 1 The Brady Urological Institute, Johns Hopkins School of Medicine, 600 N. Wolfe St., Marburg Building Room 113, Baltimore, MD 21287 USA; 2 Cellular and Molecular Medicine Program, Johns Hopkins School of Medicine, Baltimore, MD 21205 USA

**Keywords:** Lipid droplets, Polyaneuploid cancer cells, Polyploid giant cancer cells, Therapy resistance, Evolution

## Abstract

Lipid droplets (LDs) are found throughout all phyla across the tree of life. Originating as pure energy stores in the most basic organisms, LDs have evolved to fill various roles as regulators of lipid metabolism, signaling, and trafficking. LDs have been noted in cancer cells and have shown to increase tumor aggressiveness and chemotherapy resistance. A certain transitory state of cancer cell, the polyaneuploid cancer cell (PACC), appears to have higher LD levels than the cancer cell from which they are derived. PACCs are postulated to be the mediators of metastasis and resistance in many different cancers. Utilizing the evolutionarily conserved roles of LDs to protect from cellular lipotoxicity allows PACCs to survive otherwise lethal stressors. By better understanding how LDs have evolved throughout different phyla we will identify opportunities to target LDs in PACCs to increase therapeutic efficiency in cancer cells.

## Introduction

The majority of eukaryotic and bacterial cells contain some amount of cytosolic lipids or structures of a similar capacity [1]. These structures are spherical in shape and consist of neutral lipid esters or lipid based polymers all bound by a monolayer phospholipid membrane [1, 2]. Cytosolic lipid inclusions were originally described as microsomes or liposomes by Altmann, Hanstein, and Wilson [3–5]. The current terminology labels these structures as lipid droplets (LDs), although they have also been called lipid bodies, adiposomes, granules, oleosomes, and oil bodies [6]. At the most basic level, LDs are simple lipid accumulations within an aqueous cellular solution [7]. LDs most likely evolved originally in microorganisms as structures for temporary stores of excess dietary lipid [1]. LDs then evolved to acquire roles as long-term carbon stores enabling organisms to survive a lack of nutrients in the environment [1]. While LDs diversify and gain functions throughout different phyla, they all still share general characteristics and structural features [1]. In multicellular organisms it has been shown that LDs have evolved to be fundamental in cellular processes such as the trafficking of lipids, proteins, and membrane material. Malfunctioning LDs and associated proteins have been implicated in many different human diseases, including type 2 diabetes, Alzheimer’s disease, Parkinson’s disease, and cancer [1].

In cancer cells, LDs have emerged as major regulators of lipid metabolism, trafficking, and signaling [2, 8]. To ensure a proper supply of LDs, cancer cells are able to upregulate de novo lipid synthesis, repurpose structural lipids via enzymatic remodeling, uptake lipids from the environment, and recycle lipids through autophagy [9]. Cancer cells utilize these LDs to ensure energy storage and redox balance, modulate autophagy, and drive membrane synthesis which can minimize stress response and promote tumor progression [9]. Cancer cells are capable of using several lipid acquisition pathways which all converge to generating a LD. LDs also act as buffers that can consolidate the various lipid fluxes and finely tune their release and distribution in the cell allowing the drive for essential processes that control cancer cell fate [2, 9].

**Fig. 1:**
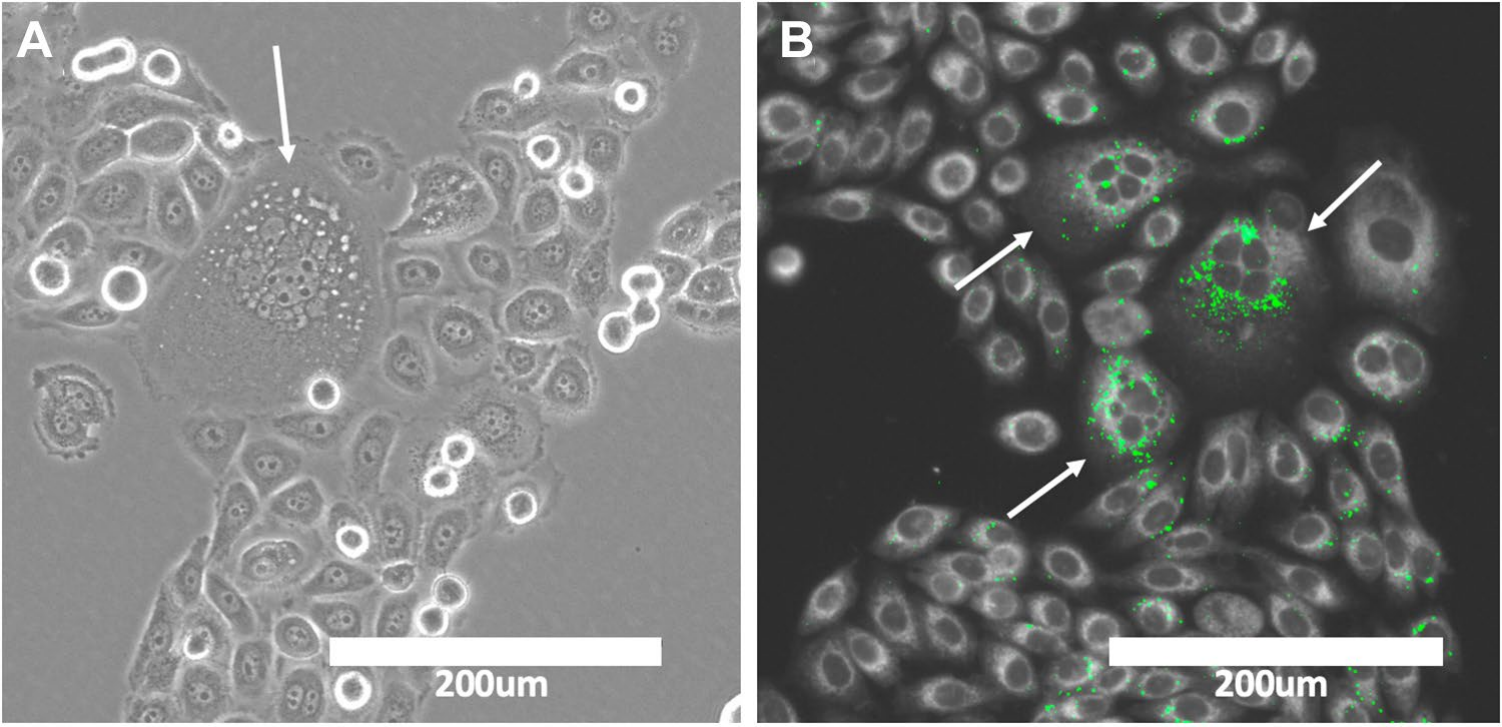
Lipid droplets in PACCs. **A** Phase contrast image of a culture of PC3 cells. Arrow pointing to LDs in a PACC. **B** Nile red staining of a culture of PC3 cells. Nile red is a stain for neutral lipid droplets. Green dots indicate LDs stained with Nile red. Arrows pointing to PACCs with LDs

New evidence has shown that the polyaneuploid cancer cell (PACC) state is a critical transition cell state in lethal cancer, including in metastasis and therapy resistance. PACCs (also referred to as polyploid giant cancer cells (PGCCs), multinucleated cancer cells, hyper diploid cells, blastomere-like cancer cells, and osteoclast-like cancer cells) are cancer cells that have undergone whole-genome multiplication (polyploidy) of their aneuploid genome (i.e., are polyaneuploid) [10–17]. PACCs have been observed in patients, in mouse models, and in cell culture across virtually all cancer types [12, 15, 16, 18–26]. PACCs form in response to many different tumor microenvironmental or applied stressors such as hypoxia, chemotherapy, or radiation and enter into a paused cell cycle state until the stress is removed [12, 15, 18–21, 24, 27–29]. The mechanism of formation of PACCs is not well understood, and it remains an active area of research to understand the protective role of the PACC state. It is interesting to note that cancer cells in the PACC state contain a higher number of LDs than their parental counterparts [30] (Fig. 1). This metabolic shift may provide these cells with a mechanism of stress resistance, including to anti-cancer therapy. Understanding how LDs evolved throughout different phyla will provide important insight into the evolutionarily conserved roles of LDs in PACCs and strategies to specifically target LDs to increase cancer cell lethality.

### Lipid droplets in phyla across the tree of life

LDs have evolved from simple energy storage organelles, to playing an important role in cell signaling and protecting the cell from cytotoxicity and death (Table 1). Learning from this evolution will provide insight of how PACCs may use LDs as a key survival mechanism under therapeutic stress. Elucidating how LDs mediate PACC viability will provide better perspective on how to target LDs in the PACC state and improve treatment outcomes in cancer patients.

**Table 1:** LD characteristics and their purported advantages in nature

Organism taxa	LD characteristics	Purported advantage	References
Archaea	• 100–500 nm diameter • Based on isoprenoid chains • Accumulate PHAs as their exclusive storage components • Structural proteins: heat shock protein	• Act exclusively as energy stores • Hyper-accumulation of LDs occurs in response to specific forms of nutrient limitation (most commonly a low carbon/nitrogen (C/N) ratio) • Accumulation of LDs is a facultative response to nutrient depletion • Accumulation of LDs marks the pause of growth and division	[1, 36]
Bacteria	Bacterial PHA droplets • 100-600 nm diameter • Components are PHAs • Structural Proteins: PHASIN (PhaP) Bacterial WE/TAG droplets •Up to 2000 nm diameter •Components are TAGs and wax esters (wax esters are frequently produced as a carbon storage material) •Structural Proteins: TadA/HBHA	• Act exclusively as energy stores • Hyper-accumulation of LDs occurs in response to specific forms of nutrient limitation (most commonly a low carbon/nitrogen (C/N) ratio) • Accumulation of LDs is a facultative response to nutrient depletion • Accumulation of LDs marks the pause of growth and division	[1, 31, 36, 80]
Protists	• Accumulate in protist and in infected host	• Several protists stimulate the formation of LDs in host cells which are then mobilized as energy sources by the parasite or pathogen	[1, 81]
Fungi	• Accumulate LDs in order to better survive as infectious agents • Function of LDs vary depending on species or developmental stage • Structural proteins: caleosin/steroleosin	• Several fungi stimulate the formation of LDs in host cells which are then mobilized as energy sources by the parasite or pathogen • LD formation commonly occurs during vegetative growth in saprophytic fungi, but LD numbers also increase during the formation of resting and reproductive structures	[1, 37]
Yeast	• Up to 1000 nm diameter • Components are TAG and sterol esters (can be one or the other exclusively, or a heterogeneous mixture) • LDs can sequester toxins	• LDs sequestering toxins allows for maintenance of organism homeostasis	[1, 36, 39]
Plants	• Components are TAGs • Some plant cells also accumulate LD-like structures called plastoglobules in their plastid organelles • The lipidic phase of plastoglobules can include TAGs, sterol esters, and various lipophilic pigments such as carotenoids • Structural proteins: Oleosin (OLE), LD- associated protein (LDAP), small rubber particle (SRP), caleosin/steroleosin	• LDs can act as energy stores • LDs can act as a buffer to take up and/or release acyl moieties to maintain cellular homeostasis	[1]
Drosophila	• 1.5% of the expressed genome is implicated in LD function (totaling ~ 370 genes) • The most common neutral lipid in insect LDs is TAG, although SEs may be present in some tissues	• *Drosophila* development is characterized by alternating TAG synthesis and mobilization phases, which cause a dramatic increase of body fat content during larval development followed by a threefold reduction during metamorphosis	[1, 44]
Mammals	• 100 nm–100um in diameter • More enzymes to assist with formation and breakdown of LDs • Generate LDs from many different pathways	• Inhibiting LD formation is difficult due to the multiple pathways utilized to generate LDs	[1, 46–49]
Cancer Cells	• Generate LDs from many different pathways • Components are TAGs and sterol esters • Accumulation of LDs	• LD accumulation has been implicated in cancer cell proliferation, resistance to death, and aggressiveness	[1, 8]
Polyaneuploid Cancer Cells	•High level of LDs	•Energy stores •Protect from lipotoxicity	[54]

#### Archaea

Archaea are a domain of single cell organisms that lack a cell nucleus. These microorganisms’ membrane lipids differ widely from bacteria and eukaryotes as they are composed of glycerol ether lipids rather than glycerol esters [1]. Additionally, archaea do not synthesize fatty acyl esters which are the most common components of LDs in other phyla. Instead, archaeal lipids are based on isoprenoid chains. All known LD-containing archaeal species are capable of accumulating a range of polymeric lipids, the most common being polyhydroxyalkanoates (PHAs). Additionally, LDs in archaea appear to act exclusively as energy stores (Table 1). A large accumulation of lipids can occur in response to specific forms of nutrient limitations (most commonly a low carbon/nitrogen ratio) [1]. These instances of accumulated lipids frequently mark a stressful period leading to the interruption of growth and division, and the entry of these unicellular organisms into a non-proliferative state [1].

#### Bacteria

Bacteria constitute a large domain of prokaryotic unicellular microorganisms. These microorganisms have cell walls but lack an organized nucleus. Bacteria differ from archaeal prokaryotes as they contain peptidoglycan in their cell walls, and their membranes are lipid bilayers compared to the archaeal membranes which can be a lipid bilayer or monolayer. Additionally, bacteria contain fatty acids on their cell membranes while archaea contain phytanyl. Similar to archaea, the majority of bacteria store carbon in the form of PHAs within LDs. However, bacteria are the only known prokaryote to have evolved the ability to also accumulate triacylglycerols (TAG) or wax esters within their LDs [1]. The highest levels of TAG accumulation in bacteria have been reported in mainly nocardioforms such as *Mycobacterium*, *Nocardia*, *Rhodococcus*, *Micromonospora*, *Dietzia*, *Gordonia,* and some streptomycetes [31, 32]. LDs in bacteria are used exclusively as energy stores (Table 1). When hydrocarbonoclastic bacteria lack suitable growth substrates they will enter a dormant phase and live off their accumulated LD reserves until a suitable growth substrate is reintroduced in the environment [33].

#### Protists

Protists are any eukaryotic single-celled organism that is not an animal, plant, or fungus. A large variety of protists act as parasites or pathogens. The ability to accumulate cytosolic LDs is key for many protists’ success as infectious agents, an example is the malarial parasite *Plasmodium falciparum* [1]. An essential factor for the proliferation of *P. falciparum* within infected human erythrocytes is the ability to accumulate and mobilize large amounts of TAGs [34]. Most of the host-derived acyl groups get transferred to the parasite and accumulate in the cytosol as TAGs are released into the infected erythrocyte. The sudden release of fatty acids in the erythrocyte causes membrane lysis and cell rupture, resulting in the release of merozoites that are now capable of infecting new cells [34]. Therefore, in *P. falciparum*, LDs have two functions: a nutrient source, and a mechanism to enable *P. falciparum* cells to escape from host cells and enter the next phase of their life cycle (Table 1) [1, 34].

#### Fungi

Fungi are eukaryotic, heterotrophic organisms that contain chitin in their cell walls and acquire food by absorbing dissolved molecules. Similar to other eukaryotes, fungal LDs appear to arise from the endoplasmic reticulum [35]. The main protein involved in LD biogenesis in fungi is the fat storage inducing transmembrane protein (FIT) [36]. Most fungi have cytosolic LDs, but the function of these LDs can vary according to species, developmental stage, or environmental conditions [1]. For example, LDs typically form during vegetative growth in saprophytic fungi (e.g., mushrooms, mold). However, LDs can also increase during the formation of their reproductive structures [1, 37]. Throughout evolution, fungal LDs have moved from only functioning as energy stores to also functioning to trap toxins within the organism to prevent it from harm (Table 1). In the endolichenic fungus *Phaeosphaeria* sp., phototoxic perylenequinones (PQs) are sequestered in LDs following exposure to light irradiation, providing resistance to the phototoxins, and the survival of the fungus [38]. This evolved trait allows fungi to utilize LDs in more than one way, improving their chances of survival in stressful conditions.

#### Yeast

Yeast are eukaryotic unicellular microorganisms. This relatively simple organism has been shown to share many features of LD organization and function found in more complex eukaryotic organisms such as mammals [1, 39]. Yeast LDs can contain either TAGs or sterol esters exclusively, or a mixed composition of the two [39]. LDs in yeast cells have many roles including being energy stores, functioning as a hub for the regulatory network that modulates TAG and SE formation in the endoplasmic reticulum, and fatty acid oxidation in peroxisomes. If LD formation is blocked in *S. cerevisiae,* exogenous fatty acids become toxic when taken up by the cells [40]. This indicates that one of the constitutive functions of these LDs is to maintain intracellular lipid homeostasis by reducing the fatty acid toxicity via sequestration of the excess acyl groups [1, 40]. The evolution of LDs to function and sequester fatty acids allows yeast cells the capability to survive in stressful situations they may have otherwise perished in (Table 1).

#### Plants

Plants are mainly multicellular photosynthetic eukaryotes in the kingdom Plante. All major groups of plants, from unicellular algae to more complex multicellular flowering plants, are capable of producing LDs in some of their cells or tissues [1]. The mechanism of LD formation in plant cells is similar to that of animal cells as the accumulation of TAGs in the endoplasmic reticulum is followed by the release of small LDs that mature into larger LDs in the cytoplasm [41]. Simple plants like algae that fall in the kingdom Plante range from small unicellular organisms to complex multicellular seaweeds. Many algal species are capable of accumulating a high amount of cytosolic TAG filled LDs as storage reserves in response to stress [42]. These LDs can make up as much as 86% of the cell dry weight, allowing the plant to survive off these stores for long periods of time [1]. In more complex plants, LD accumulation was first thought to be confined to specific tissues with the singular function of energy storage. However, new evidence has shown the presence of dynamic LDs that do not accumulate as long-term lipid stores. In most cases, LDs in plants will accumulate TAGs (one known species, oilseed, accumulates fluid wax esters instead). In addition to cytosolic LDs, some plants accumulate structures similar to LDs known as plastoglobules in their plastid organelles. Platoglobules have a lipidic phase which can include TAGs, sterol esters, and various lipophilic pigments. In terrestrial plants, there is evidence showing that cytosolic TAG rich LDs are mainly detected in the leaf mesophyll cells. Slocombe and colleagues noted that TAG accumulation in leaves is increased following a manipulation of fatty acid breakdown and lipid synthesis indicating that cytosolic LDs in leaves could be acting as a buffer to take up or release acyl moieties in order to maintain metabolic homeostasis in cells [43]. From energy stores in algae to buffering cell toxicity in higher plants, LDs prove themselves as important organelles in plant survival (Table 1).

#### Drosophila

*Drosophila melanogaster* has been acknowledged as an invertebrate model organism for observing core functions of LDs [1, 44]. Most Drosophila LDs are prominent organelles in tissues dedicated to TAG (neutral lipid) storage and breakdown [44]. Neutral lipids can be transported between storage sites as lipoprotein particles [44]. Drosophila lipoprotein particles mainly are composed of diacylglycerol (DAG) but can also be composed of lipophorins, phospholipids, and transport lipids [44]. This unique composition suggests that storage TAGs are deacylated before they are loaded onto pre-formed lipoprotein particles [44]. Lipoprotein particles can then be unloaded at their intended site through interactions with lipophorin receptors in an endocytosis-independent manner [44]. Beyond their role in lipid transport, lipoprotein particles also serve important signaling functions in flies (i.e., modulation of the wingless [WNT] and hedgehog [Hh] morphogen activities). The Drosophila LD proteome is similar to that of mammals, indicating evolutionarily conserved functions [1]. This makes Drosophila a good model to investigate LD malfunction in human diseases such as metabolic syndrome and lipodystrophy [1]. LDs in Drosophila serve many purposes allowing these organisms to flourish in many environments while dysregulation of LD formation or utilization can be extremely detrimental (Table 1).

#### Mammals

In mammals, the majority of LDs are found in adipose tissue, liver, and muscle [45]. Mammalian cells have similar basic LD systems as other animal groups. However, in higher mammals, such as placental mammals, basic LD mechanisms such as energy storage are accompanied by additional layers of complexity [1]. These complex mechanisms have been adapted throughout the evolution of these long-lived group of eukaryotes [1]. Mammalian LDs are involved in lipid transport, lipid synthesis, lipid degradation, and protein degradation [45] (Table 1). At the metabolic level, mammals have a larger variety of key enzymes to assist with LD formation (DAG acyltransferase) [46] and LD turnover/breakdown (lipases) [47–49]. Having a larger variety of enzymes for LD formation and degradation allows for one pathway to compensate if another pathway is dysregulated, still ensuring proper LD operations. Conversely, this indicates that to efficiently halt LD formation or breakdown, targeting multiple pathways may be necessary.

#### Cancer cells

One of the hallmarks of cancer is metabolic reprogramming [50, 51]. Beyond the classical metabolomic changes highlighted by the Warburg effect, research has pointed to the metabolism of lipids as critical for tumorigenesis [2, 9, 51, 52]. Cancer cells have remarkable metabolic plasticity that allows them to adapt to adverse conditions and drives their resistant and metastatic potential [50]. Even with remarkable plasticity, cancer cells need a mechanism of survival as they are constantly faced with various stressors (e.g., hypoxia of the tumor microenvironment or applied anti-cancer chemotherapy). When stressed, a subset of cancer cells undergo polyploidization to enter a transient PACC state [16, 18, 29, 53]. Cells in the PACC state contain a higher number of LDs than the cancer cells they are derived from (Fig. 1) [30, 54]. As PACCs are hypothesized to be mediators of tumorigenesis, metastasis, and resistance, understanding their LD biology may be crucial in learning how to target them [18].

LDs have emerged as novel regulators of many metabolic processes in cancer cells [2, 9]. LDs are dynamic organelles capable of responding to nutrient fluctuations and environmental stressors. These responses allow the cell to control trafficking, storage, and use of the neutral lipids within the LDs [2, 55–59]. An increase in LDs can affect major cancer hallmarks including cell growth, proliferation, metabolism, migration, inflammation, and immunity [2, 8, 57, 60–62]. LDs are also associated with protein turnover, gene transcription, and nuclear function [9]. LDs act as switches that can coordinate lipid trafficking and consumption for various purposes. In healthy cells, free fatty acids are absorbed directly and used for energy, so their lipid synthesis pathway is typically inactive. In contrast, the lipid synthesis pathway in cancer cells is activated while the lipid breakdown processes are limited. When under metabolic stress, different cancer types have demonstrated different lipid metabolism responses [63, 64]. Under metabolic stress, some cancer cells can upregulate FA synthesis while other cancer cells increase intake of exogenous FAs from the microenvironment for survival [2, 63, 65, 66]. Cancer cells capable of acquiring extracellular FAs or adapting intracellular mechanisms for lipid generation, mobilization, and recycling have a higher chance of survival [2]. Some aggressive cancer types use opportunistic mechanisms of FA acquisition and increase their capacity for FA accumulation in LDs [67–69]. However, too high levels of free fatty acids or other lipids in a cell can lead to lipotoxicity and cell death unless sequestered. LDs are capable of sequestering toxic lipids to reduce cellular toxicity [1, 2]. LDs can also affect drug efficacy by altering cellular distribution and activation of lipophilic anti-cancer agents (i.e., cisplatin, ponatinib), rendering some anti-cancer therapies ineffective [70, 71]. Learning more about LD function and biogenesis may give insight on how to target them to increase toxicity in cancer cells and eventually lead to their death.

## Discussion: understanding LD evolution may provide insight in how to target them in PACCs

LDs are diverse organelles that allow organisms to better survive stress in their environment. Although many mechanistic details surrounding LDs remain elusive, there has been progress toward understanding their biogenesis and many of the proteins involved in this process. Cancer cells have been shown to accumulate LDs which drive tumor aggressiveness and chemotherapy resistance. Additionally, we and others have shown that the transient PACC state has greater accumulation of LDs than the typical cancer cell [30, 54]. As PACCs are hypothesized to be lethal mediators of cancer metastasis and therapy resistance, understanding LD function in PACCs is crucial. By better understanding the mechanisms controlling the formation and dynamics of LDs in PACCs we may ultimately be able to better understand the pathogenesis of cancer. Looking at the evolution of LD function gives insight into how LDs have become integral to cell biology and how targeting them in cancer, specifically in PACCs, may lead to lower cancer lethality in patients.

While LDs initially evolved as pure energy stores in prokaryotic organisms such as archaea and bacteria, they have gained many functions and constituents across the multiple kingdoms of life. For example, when under stress, prokaryotic organisms can accumulate LDs and then enter into a non-proliferative state and live of these LDs as energy stores until nutrients are available again. In yeast, LDs sequester abundant free fatty acids that would otherwise cause toxicity in these cells. Mammalian LDs can be formed from many different pathways, providing critical redundancy for maintaining LD formation. All of these characteristics are key survival mechanisms that cancer cells are also able to access.

Prokaryotic organisms utilize LDs exclusively as energy stores. In times of stress these prokaryotes accumulate LDs which can be used as energy reserves while nutrients are low. Additionally, when prokaryotes have this hyper-accumulation of LDs, they shut down proliferation in order to lower metabolic functions until nutrients are available again. These mechanisms may be similar in PACCs. Even in the absence of applied chemotherapy, PACCs under standard conditions visibly have a higher amount of LDs compared to adjacent non-polyaneuploid cancer cells in the same culture. Upon induction with chemotherapy, PACCs accumulate an ever higher level of LDs [30]. These cells then enter into a non-proliferative state that persists until the cells are completely recovered from the stress of chemotherapy [72, 73]. PACCs could potentially be utilizing these accumulated LDs as energy stores instead of taking up potentially toxic nutrients from the environment. By entering a non-proliferative state and containing accumulated LDs, prokaryotes and PACCs have a higher chance at surviving a potentially otherwise uninhabitable environment.

**Fig. 2:**
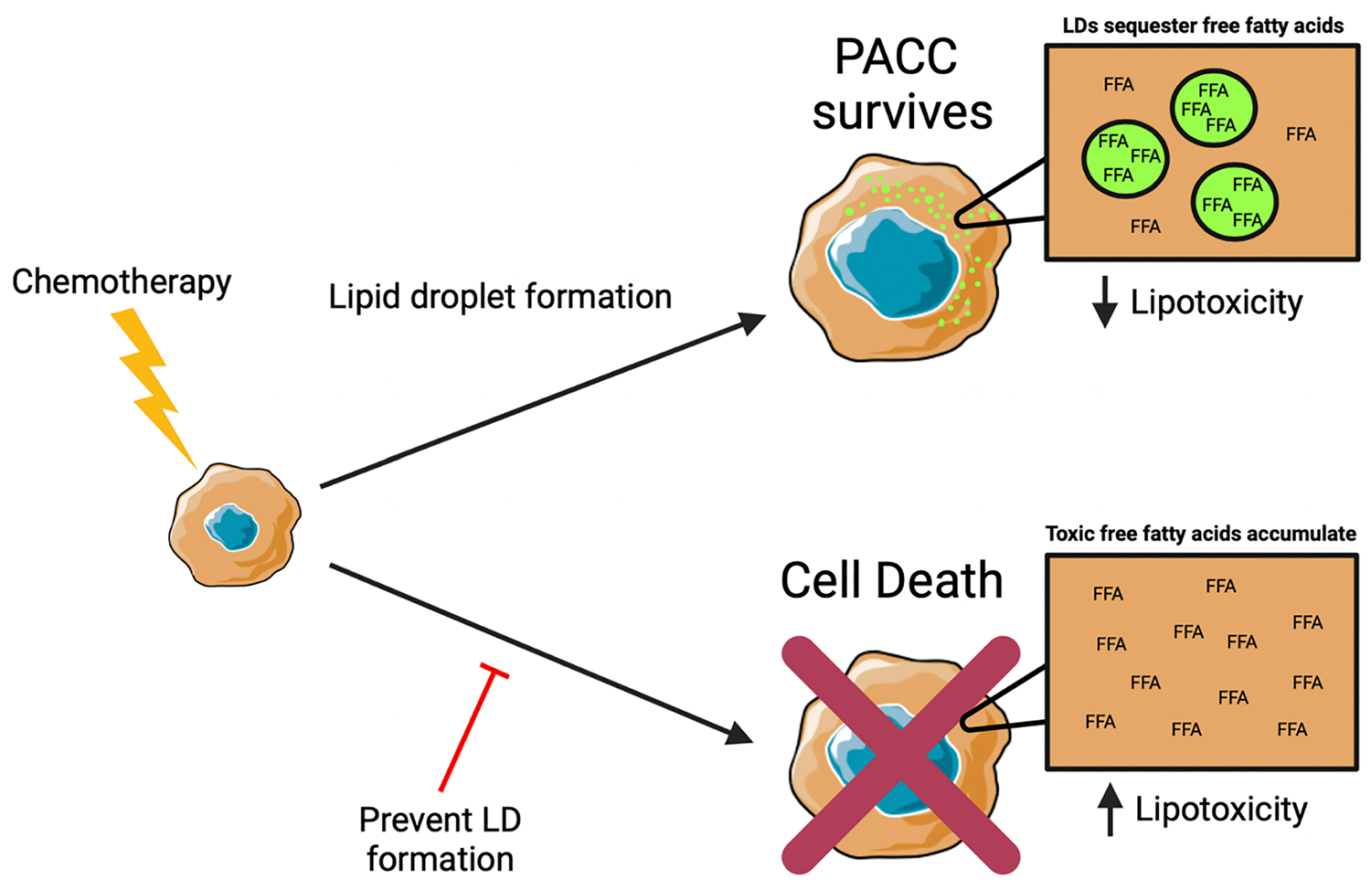
Polyaneuploid cancer cells utilize LDs to protect from lipotoxicity. Chemotherapy is applied to a cancer cell. Surviving cancer cells can become a PACC that is filled with free fatty acids due to the potential upregulation of lipid synthesis and fatty acid synthesis. If free fatty acids are able to be sequestered in LDs, the PACC is able to survive. If the PACC cannot produce LDs and sequester free fatty acids, the PACC will die due to lipotoxicity

The eukaryotic organism, S. cerevisiae utilizes LDs both as energy stores and to prevent cellular toxicity. It has been shown that if LD formation in S. cerevisiae is blocked the organisms will fill with exogenous fatty acids that are toxic when at a high level [46, 47]. This indicates that LDs sequestering free fatty acids lowers cellular toxicity and maintains cellular homeostasis. Similarly, when a cancer cell population is treated with chemotherapy the vast majority of the cancer cells die due to irreparable damage. However, a subset of cancer cells survive by accessing the PACC state and an accompanying accumulation of LDs. The high level of LD accumulation may provide a mechanism of survival for PACCs by sequestering the free fatty acids or other various toxins in the cell to survive (Fig. 2). This adaption may allow PACCs the capability of resisting many different classes of chemotherapeutic treatments as seen in the literature [29, 74, 75].

Mammals have a larger variety of key enzymes that assist with LD formation and LD breakdown [46, 47]. The advantage to having this larger variety of enzymes is that many different pathways can generate a fatty acid or sterol ester that can then be packaged into a LD, allowing for one pathway to compensate for another if it becomes dysregulated. Cancer cells also have multiple ways of accessing fatty acids for LDs. They can undergo TAG synthesis, fatty acid synthesis, or uptake fatty acids from the environment. These compensatory pathways represent a challenge for targeting LDs, as multiple different pathways may need to be inhibited simultaneously.

It has become increasingly important in the last decade to study and understand PACCs. Once thought to be artifact, these cells have now shown to be key in tumor aggressiveness and resistance [10, 12, 14, 18–20, 23, 53, 76]. Forming from cancer cells under stress, the PACC state is known to be non-proliferative and contain high levels of LDs (Fig. 1). Targeting LD formation and lipid metabolism to increase PACC lethality may be the next step to decreasing patient lethality. One study demonstrated that DGAT1 in glioblastoma cells is capable of protecting tumors from lipotoxicity, labeling it as a potential metabolic target for cancer therapy [77]. Studies have also shown that the increase of nitric oxide (NO) levels in LDs can protect tumor cells and reduce the efficiency of chemotherapeutic drugs [78]. Varying studies have been focusing on utilizing NO inhibitors to effectively improve the effect of chemotherapeutic reagents [78]. Additional studies have shown that the chemotherapeutic agents, 5-fluorouracil, and oxaliplatin induce lysophosphatidylcholine acyltransferase 2 (LPCAT2) dependent LD accumulation [79]. LPCAT2-driven LD biogenesis was shown to protect cancer cells from this chemotherapy-induced ER stress and cell death in vivo and in vitro [79]. This study elucidated LPCAT2 as another potential target to prevent LD formation. Last, a recent study has shown that by activating the liver X receptor alpha (LXRa) and inhibiting the Raf-1-SCD1 protein complex in liver carcinomas, an intracellular accumulation of saturated free fatty acids occurs and leads to lethal lipotoxicity in these tumor cells. Overall, targeting lipids could potentially eliminate PACCs entirely.

## Conclusion

The past few decades have introduced knowledge of the various roles of LDs in cellular function throughout a range of biological organisms. LDs have been found to be highly dynamic in short term processes such as signaling, as well as in longer term processes like energy storage. In higher eukaryotes, LDs have evolved new functions with roles in developmental processes, environmental responses, pathogenesis, and implications in disease which can affect the organism as a whole. LD dysfunction in humans has implications in serious diseases including cancer. The increase of LDs in cancer cells implicates higher aggressiveness and chemotherapy resistance. PACCs, containing an even higher amount of LDs than typical cancer cells have an additional advantage for LD-mediated survival. Targeting LD formation to increase chemotherapy efficacy represents a promising candidate for eliminating the lethal PACC state and reducing therapy resistance.

## Data Availability

Not applicable. Not applicable.

## References

[1] Murphy DJ (2012). The dynamic roles of intracellular lipid droplets: from archaea to mammals. Protoplasma.

[2] Petan T, Jarc E, Jusović M (2018). Lipid droplets in cancer: guardians of fat in a stressful world. Molecules.

[3] Altmann R (1890). Die Elementarorganismen und ihre Beziehungen zu den Zellen.

[4] J. Hanstein, “Ueber die Gestaltungsvorgange in den Zellkerne bei der Theilung der Zellen,” *Botan Abhandl Morphol Physiol Bonn*, 1880.

[5] E. B. Wilson, *The cell in development and inheritance*. The Macmillan company, 1896, pp. 1–400. [Online]. Available: https://www.biodiversitylibrary.org/item/99997

[6] Martin S, Parton RG (2006). Lipid droplets: a unified view of a dynamic organelle. Nat Rev Mol Cell Biol.

[7] Thiam AR, Farese RV, Walther TC (2013). The biophysics and cell biology of lipid droplets. Nat. Rev. Mol. Cell Biol..

[8] Cruz ALS, Barreto EA, Fazolini NPB, Viola JPB, Bozza PT (2020). Lipid droplets: platforms with multiple functions in cancer hallmarks. Cell Death Dis.

[9] Petan T (2020). Lipid droplets in cancer.

[10] Pienta KJ, Hammarlund EU, Axelrod R, Brown JS, Amend SR (2020). Poly-aneuploid cancer cells promote evolvability, generating lethal cancer. Evol Appl.

[11] Mosieniak G, Sikora E (2010). Polyploidy: the link between senescence and cancer. Curr Pharm Des.

[12] Erenpreisa J (2008). Endopolyploidy in irradiated p53-deficient tumour cell lines: persistence of cell division activity in giant cells expressing aurora B- kinase. Cell Biol Int.

[13] Erenpreisa JA, Cragg MS, Fringes B, Sharakhov I, Illidge TM (2000). Release of mitotic descendants by giant cells from irradiated burkitt’s lymphoma cell lines. Cell Biol Int.

[14] Fei F (2015). The number of polyploid giant cancer cells and epithelial-mesenchymal transition-related proteins are associated with invasion and metastasis in human breast cancer. J Exp Clin Cancer Res.

[15] Chen J (2019). Polyploid giant cancer cells (PGCCs): the evil roots of cancer. Curr Cancer Drug Targ.

[16] Zhang S, Mercado-Uribe I, Xing Z, Sun B, Kuang J, Liu J (2014). Generation of cancer stem-like cells through the formation of polyploid giant cancer cells. Oncogene.

[17] Zhang L, Wu C, Hoffman RM (2015). Prostate cancer heterogeneous high-metastatic multi-organ-colonizing chemo-resistant variants selected by serial metastatic passage in nude mice are highly enriched for multinucleate giant cells. PLoS ONE.

[18] Amend SR (2019). Polyploid giant cancer cells: Unrecognized actuators of tumorigenesis, metastasis, and resistance. Prostate.

[19] Mirzayans R, Andrais B, Murray D (2018). Roles of polyploid/multinucleated giant cancer cells in metastasis and disease relapse following anticancer treatment. Cancers.

[20] Illidge TM, Cragg MS, Fringes B, Olive P, Erenpreisa JA (2000). Polyploid giant cells provide a survival mechanism for p53 mutant cells after DNA damage. Cell Biol Int.

[21] Mittal K (2017). Multinucleated polyploidy drives resistance to docetaxel chemotherapy in prostate cancer. Br J Cancer.

[22] Ogden A, Rida PCG, Knudsen BS, Kucuk O, Aneja R (2015). Docetaxel-induced polyploidization may underlie chemoresistance and disease relapse. Cancer Lett.

[23] Zhang S, Mercado-Uribe I, Hanash S, Liu J (2013). iTRAQ-based proteomic analysis of polyploid giant cancer cells and budding progeny cells reveals several distinct pathways for ovarian cancer development. PLoS ONE.

[24] Puig P-E (2008). Tumor cells can escape DNA-damaging cisplatin through DNA endoreduplication and reversible polyploidy. Cell Biol Int.

[25] Niu N, Mercado-Uribe I, Liu J (2017). Dedifferentiation into blastomere-like cancer stem cells via formation of polyploid giant cancer cells. Oncogene.

[26] Virchow R (1989). As based upon physiological and pathological histology. Nutr Rev.

[27] Lopez-Sánchez LM (2014). CoCl2, a mimic of hypoxia, induces formation of polyploid giant cells with stem characteristics in colon cancer. PLoS ONE.

[28] Makarovskiy AN, Siryaporn E, Hixson DC, Akerley W (2002). “Survival of docetaxel-resistant prostate cancer cells in vitro depends on phenotype alterations and continuity of drug exposure”. Cell Mol Life Sci.

[29] Lin K-C (2019). The role of heterogeneous environment and docetaxel gradient in the emergence of polyploid, mesenchymal and resistant prostate cancer cells. Clin Exp Metastasis.

[30] Sirois I (2019). A unique morphological phenotype in chemoresistant triple-negative breast cancer reveals metabolic reprogramming and PLIN4 expression as a molecular vulnerability. Mol Cancer Res.

[31] Alvarez H, Steinbüchel A (2002). Triacylglycerols in prokaryotic microorganisms. Appl Microbiol Biotechnol.

[32] Kosa M, Ragauskas AJ (2011). Lipids from heterotrophic microbes: advances in metabolism research. Trends Biotechnol.

[33] Kalscheuer R (2007). Analysis of storage lipid accumulation in Alcanivorax borkumensis: evidence for alternative triacylglycerol biosynthesis routes in bacteria. J Bacteriol.

[34] Palacpac NMQ (2004). Developmental-stage-specific triacylglycerol biosynthesis, degradation and trafficking as lipid bodies in *Plasmodium falciparum*-infected erythrocytes. J Cell Sci.

[35] Schneider EF, Seaman WL (1977). Ontogeny of lipid bodies in the endoplasmic reticulum of Fusarium sulphureum. Can J Microbiol.

[36] Lundquist PK, Shivaiah K-K, Espinoza-Corral R (2020). Lipid droplets throughout the evolutionary tree. Prog Lipid Res.

[37] Murphy DJ. “Plant lipids: biology, utilisation and manipulation.,” *Plant lipids: biology, utilisation and manipulation.*, 2005. [Online]. https://www.cabdirect.org/cabdirect/abstract/20053054916 Accessed 07 Jun 2021

[38] Chang W (2015). Trapping toxins within lipid droplets is a resistance mechanism in fungi. Sci Rep.

[39] Czabany T (2008). Structural and biochemical properties of lipid particles from the yeast *Saccharomyces **cerevisiae**. J Biol Chem.

[40] Lockshon D, Surface LE, Kerr EO, Kaeberlein M, Kennedy BK (2007). The Sensitivity of yeast mutants to oleic acid implicates the peroxisome and other processes in membrane function. Genetics.

[41] Gidda SK (2011). Hydrophobic-domain-dependent protein-protein interactions mediate the localization of GPAT enzymes to ER subdomains. Traffic.

[42] Wang ZT, Ullrich N, Joo S, Waffenschmidt S, Goodenough U (2009). Algal lipid bodies: stress induction, purification, and biochemical characterization in wild-type and starchless Chlamydomonas reinhardtii. Eukaryot Cell.

[43] Slocombe SP (2009). Oil accumulation in leaves directed by modification of fatty acid breakdown and lipid synthesis pathways. Plant Biotechnol J.

[44] Kühnlein RP (2011). The contribution of the drosophila model to lipid droplet research. Prog Lipid Res.

[45] Thiele C, Penno A, Bradshaw RA, Stahl PD (2016). Lipid droplets. Encyclopedia of cell biology.

[46] Harris CA (2011). DGAT enzymes are required for triacylglycerol synthesis and lipid droplets in adipocytes[S]. J Lipid Res.

[47] Jenkins CM, Mancuso DJ, Yan W, Sims HF, Gibson B, Gross RW (2004). Identification, cloning, expression, and purification of three novel human calcium-independent phospholipase A2 family members possessing triacylglycerol lipase and acylglycerol transacylase activities*. J Biol Chem.

[48] Osuga J (2000). Targeted disruption of hormone-sensitive lipase results in male sterility and adipocyte hypertrophy, but not in obesity. Proc Natl Acad Sci.

[49] Zimmermann R (2004). Fat mobilization in adipose tissue is promoted by adipose triglyceride lipase. Science.

[50] Hanahan D, Weinberg RA (2011). Hallmarks of cancer: the next generation. Cell.

[51] Pavlova NN, Thompson CB (2016). The emerging hallmarks of cancer metabolism. Cell Metab.

[52] Ward PS, Thompson CB (2012). Metabolic reprogramming: a cancer hallmark even warburg did not anticipate. Cancer Cell.

[53] Pienta KJ, Hammarlund EU, Austin RH, Axelrod R, Brown JS, Amend SR (2020). Cancer cells employ an evolutionarily conserved polyploidization program to resist therapy. Semin Cancer Biol.

[54] Kostecka LG, Pienta KJ, Amend SR (2021). Polyaneuploid cancer cell dormancy: lessons from evolutionary phyla. Front Ecol Evol.

[55] Farese RV, Walther TC (2009). Lipid droplets finally get a little R-E-S-P-E-C-T. Cell.

[56] Jarc E, Petan T (2020). A twist of FATe: lipid droplets and inflammatory lipid mediators. Biochimie.

[57] Koizume S, Miyagi Y (2016). Lipid droplets: a key cellular organelle associated with cancer cell survival under normoxia and hypoxia. Int J Mol Sci.

[58] Krahmer N, Farese RV, Walther TC (2013). Balancing the fat: lipid droplets and human disease. EMBO Mol Med.

[59] Olzmann JA, Carvalho P (2019). Dynamics and functions of lipid droplets. Nat Rev Mol Cell Biol.

[60] Attané C, Muller C (2020). Drilling for oil: tumor-surrounding adipocytes fueling cancer. Trends in Cancer.

[61] Currie E, Schulze A, Zechner R, Walther TC, Farese RV (2013). Cellular fatty acid metabolism and cancer. Cell Metab.

[62] den Brok MH, Raaijmakers TK, Collado-Camps E, Adema GJ (2018). Lipid droplets as immune modulators in myeloid cells. Trends Immunol.

[63] Munir R, Lisec J, Swinnen JV, Zaidi N (2019). Lipid metabolism in cancer cells under metabolic stress. Br J Cancer.

[64] Germain N, Dhayer M, Boileau M, Fovez Q, Kluza J, Marchetti P (2020). Lipid metabolism and resistance to anticancer treatment. Biology (Basel).

[65] Ackerman D, Simon MC (2014). Hypoxia, lipids, and cancer: surviving the harsh tumor microenvironment. Trends Cell Biol.

[66] Michalopoulou E, Bulusu V, Kamphorst JJ (2016). Metabolic scavenging by cancer cells: when the going gets tough, the tough keep eating. Br J Cancer.

[67] Pucer A, Brglez V, Payré C, Pungerčar J, Lambeau G, Petan T (2013). Group X secreted phospholipase A2 induces lipid droplet formation and prolongs breast cancer cell survival. Mol Cancer.

[68] Jarc E, Kump A, Malavašič P, Eichmann TO, Zimmermann R, Petan T (2018). Lipid droplets induced by secreted phospholipase A2 and unsaturated fatty acids protect breast cancer cells from nutrient and lipotoxic stress. Biochim et Biophys Acta (BBA)—Mol Cell Biol Lipids.

[69] Bensaad K (2014). Fatty acid uptake and lipid storage induced by HIF-1α contribute to cell growth and survival after hypoxia-reoxygenation. Cell Rep.

[70] Chen L (2020). Targeting lipid droplet lysophosphatidylcholine for cisplatin chemotherapy. J Cell Mol Med.

[71] Englinger B (2020). Lipid droplet-mediated scavenging as novel intrinsic and adaptive resistance factor against the multikinase inhibitor ponatinib. Int J Cancer.

[72] Pienta KJ, Hammarlund EU, Brown JS, Amend SR, Axelrod RM (2021). Cancer recurrence and lethality are enabled by enhanced survival and reversible cell cycle arrest of polyaneuploid cells. Proc Natl Acad Sci.

[73] Erenpreisa J, Cragg MS (2013). Three steps to the immortality of cancer cells: senescence, polyploidy and self-renewal. Cancer Cell Int.

[74] Xuan B, Ghosh D, Cheney EM, Clifton EM, Dawson MR (2018). Dysregulation in actin cytoskeletal organization drives increased stiffness and migratory persistence in polyploidal giant cancer cells. Sci Rep.

[75] Tagal V, Roth MG (2021). Loss of aurora kinase signaling allows lung cancer cells to adopt endoreplication and form polyploid giant cancer cells that resist antimitotic drugs. Cancer Res.

[76] Zheng L (2012). Polyploid cells rewire DNA damage response networks to overcome replication stress-induced barriers for tumour progression. Nat Commun.

[77] Cheng X, Geng F, Guo D (2020). DGAT1 protects tumor from lipotoxicity, emerging as a promising metabolic target for cancer therapy. Mol Cell Oncol.

[78] Ye M (2020). Deep imaging for visualizing nitric oxide in lipid droplets: discovering the relationship between nitric oxide and resistance to cancer chemotherapy drugs. Chem Commun.

[79] Cotte AK (2018). Lysophosphatidylcholine acyltransferase 2-mediated lipid droplet production supports colorectal cancer chemoresistance. Nat Commun.

[80] Wältermann M, Steinbüchel A (2005). Neutral lipid bodies in prokaryotes: recent insights into structure, formation, and relationship to eukaryotic lipid depots. J Bacteriol.

[81] Vallochi AL, Teixeira L, Oliveira KS, Maya-Monteiro CM, Bozza PT (2018). Lipid Droplet, a key player in host-parasite interactions. Front Immunol.

